# Message to the caregivers: communicating to patients on hand hygiene improves your practices!

**DOI:** 10.1186/2047-2994-4-S1-P292

**Published:** 2015-06-16

**Authors:** D Verjat-Trannoy, M-A Ertzscheid, S Monier, N Jouzeau, D Zaro-Goni, P Astagneau, From behalf the GHRYM workshop

**Affiliations:** 1 French Healthcare Associated Infection Control Network (CClin-Arlin), France

## Introduction

Since 2012, in order to improve caregivers-to-patient information and awareness about hand hygiene (HH), a communication campaign has been launched in healthcare facilities (HCF). The patients have been systematically informed of practices which are recommended during cares.

## Objectives

To identify and characterize changes in caregivers HH practices after patient information.

## Methods

In 2013 and 2014, an evaluation of caregivers HH changes was proposed to voluntary HCF. In the participating services, reporting was organized for caregivers using standardized questionnaire including different HH possible changes: better HH compliance, systematic use of handrub products, withdrawing hand jewelry.

## Results

A total of 22 HCF participated in the study. The questionnaire was completed by 269 caregivers (115 nurses, 105 nurses aides and 49 others professions), of whom 32% declared no need to change because they were already following recommendations and 49% reported having changed at least one practice. No change in HH practices was declared by 18% of the caregivers. Among caregivers who changed their HH practices (n=132), 63% (n=83) used more systematically handrub products, 58% (n=76) increased their HH compliance and 34% (n= 45) withdrew their hand jewelry. The global proportion of HH changes was not different in nurses and aides but differences were found by profession for the reported changes.

## Conclusion

After the patient information on hand hygiene, half of participating caregivers have declared a change in their HH practices. However, the sustainability of the changes remains to be verified. This study could be supplemented by observations of practice or a measure of the hand-rub consumption to confirm these encouraging results.

## Disclosure of interest

None declared.

